# Review of the *Bobekia*-group (Braconidae, Alysiinae, Alysiini), with description of a new genus and a new subgenus

**DOI:** 10.3897/zookeys.926.47270

**Published:** 2020-04-13

**Authors:** Ruo-Nan Zhang, Cornelis van Achterberg, Xiao-Xia Tian, Jiang-Li Tan

**Affiliations:** 1 Shaanxi Key Laboratory for Animal Conservation/Key Laboratory of Resource Biology and Biotechnology in Western China, Ministry of Education, College of Life Sciences, Northwest University, Xi’an 710069, China; 2 State Key Laboratory of Rice Biology and Ministry of Agriculture Key Lab of Agricultural Entomology, Institute of Insect Sciences, Zhejiang University, Hangzhou 310058, China

**Keywords:** *
Bobekia
*, *
Bobekoides
*, *
Hovalysia
*, *
Hylcalosia
*, key, *
Neodiasta
*, new species, new genera, *
Parabobekoides
*, *
Phasmidiasta
*, *
Senwot
*, *
Separatatus
*, world revision

## Abstract

The world genera of the *Bobekia*-group of Alysiini (Braconidae: Alysiinae) are reviewed and keyed. A new genus (*Neodiasta***gen. nov.**) is proposed for *Phasmidiasta ecuadorensis* Fischer, 2006, from Ecuador. One new subgenus (*Parabobekoides***subg. nov.**; type species Separatatus (Parabobekoides) yinshani**sp. nov.** from NW China) is described and illustrated. *Neosymphanes* Belokobylskij, 1998 is a new synonym of *Bobekia* Niezabitowski, 1910 (**syn. nov.**).

## Introduction

The group of alysiine genera with sculptured second metasomal tergite, distinct dorsope in the first metasomal tergite, and a closed first subdiscal cell of the fore wing (here defined as the *Bobekia*-group of the Alysiinae, Braconidae) is comprised of eight genera worldwide, with *Phasmalysia* Tobias, 1971, as a ninth borderline genus. The lack of comprehensive keys has confused several researchers and resulted in associating Chinese species with Afrotropical genera (Wharton 2002; Zheng et al. 2013). The recent series of papers on this group (Zheng et al. 2013, 2017, 2018; Belokobylskij 2015; Yao et al. 2018a, b, 2019) and the discovery in Shaanxi (NW China) of a new species with conspicuous sexual dimorphism seems the right moment to give a revised key to all genera of this group worldwide; to describe a new genus, a new subgenus, and one new species; and to report the sexual dimorphism present in the new species.

The biology is unknown within most genera, but at least some species are putatively parasitoids of xylophilous fly larvae, because their cocoons have been found in the galleries of scilytine beetles (Wharton 1980; Belokobylskij 1998). Others seem to parasitize presumably more easily accessible hosts such as mining larvae of Agromyzidae and Muscidae (see note under *Bobekia* Niezabitowski).

## Material and methods

The specimens were collected in Malaise traps in an abandoned garden in the village of Shangluo (NW China: Shaanxi, Luonan) and directly preserved in 70% alcohol; they were later chemically treated with a mixture of 96% xylene + alcohol and amylacetate (AXA-method; van Achterberg 2009) before card-pointing. For identification of the subfamily Alysiinae, see van Achterberg (1990, 1993); for identification of the genera of Chinese Alysiini, see Zhu et al. (2017); for references, see Yu et al. (2016).

The type species of all genera were examined for the key, except for *Senwot* Wharton, which was included using information from existing literature. Morphological terminology follows van Achterberg (1988, 1993), including the abbreviations for wing venation. Measurements are taken as indicated by van Achterberg (1988): for the length and the width of a body part the maximum length and width is taken, unless otherwise indicated. The length of the mesosoma was measured from the anterior border of the mesoscutum to the apex of the propodeum and of the first tergite from the posterior border of the adductor to the medio-posterior margin of the tergite.

Observations and descriptions were made with an Opto-Edu A230903 stereomicroscope and a fluorescent lamp. Photographic images were made with the Keyence VHX-5000 digital microscope. The following acronyms are used for the depositories: **AEI** – American Entomological Institute, Utah State University (Logan); **BZL** – Biologiezentrum - Oberösterreichisches Landesmuseum (Linz); **CNC** – Canadian National Collection of Insects (Ottawa); **FAFU** – Fujian Agricultural and Forestry University (Fuzhou); **MNHN** – Muséum National d’Histoire Naturelle (Paris); **NWUX** – Department of Life Sciences, Northwest University (Xi’an); **PAN** – Muzeum i Instytut Zoologii Polskiej Akademii Nauk (Warsaw); **RMNH** – Naturalis Biodiversity Center (Leiden); **ZIL** – Zoological Institute (Lund); **ZMB** – Museum für Naturkunde (Berlin).

## Key to the world genera of the *Bobekia*-group

**Table:** 

1	Second metasomal tergite distinctly sculptured basally (Figs 9, 21, 27), dorsope distinctly developed (Figs 9, 27, 96) and first subdiscal cell of fore wing closed distally (Figs 5, 20, 36); mandible without oblique ventral carina (Figs 15, 41, 74, but present in *Phasmidiasta*; Figs 102, 103); mandible often with 4 teeth or lobes (Figs 15, 17, 41, 71); pronope present and usually medium-sized to rather large (Figs 8, 46, 116); *Bobekia*-group	**2**
–	Either second tergite smooth basally or dorsope absent or first subdiscal cell of fore wing open distally; oblique ventral carina of mandible often present and mandible usually with 3 teeth; pronope variable	**other genera of Alysiini**
2	Vein r of fore wing issued at basal 0.2–0.4 of pterostigma (Fig. 107); pterostigma parallel-sided (Fig. 107); first (= dorsal) tooth much wider than third (= ventral) tooth and dorso-apically with small to medium-sized, tooth-shaped protuberance; vein CU1b of fore wing distinctly longer than vein 3-CU1 or 3-CU1 absent, first subdiscal cell narrow and vein CU1a at level of vein CU1 (Fig. 107); vein M+CU of hind wing 0.7–1.0× as long as vein 1-M (Fig. 107); [face distinctly convex in type species, less so in Asian spp. (Fig. 111); upper valve of ovipositor convex and with small nodus (Fig. 114); clypeus triangular and acute; Afrotropical species have pterostigma more elongate than Asian spp.]; Afrotropical, Oriental	***Senwot* Wharton, 1983**
–	Vein r of fore wing issued medially from pterostigma or behind it (Figs 1, 5, 17, 20, 79, 92); pterostigma triangular or elliptical (Figs 1, 17, 40, 65), but parallel-sided or nearly so in *Neodiasta* (Fig. 79); first tooth of mandible similar to other teeth (Figs 14, 25, 39, 57), if much larger then without dorso-apical protuberance (Fig. 41, but present *in Hylcalosia ruficeps*; Fig. 71); vein CU1b of fore wing distinctly shorter than vein 3-CU1, first subdiscal cell moderately wide and vein CU1a distinctly below level of vein CU1 (Figs 5, 20, 36, 97); vein M+CU of hind wing at least 1.2× as long as vein 1-M (Figs 6, 20, 53, 65, 92), but shorter than vein 1-M in *Neodiasta* (Fig. 80)	**3**
3	Clypeus acute ventrally, triangular (Fig. 43,); first tooth of mandible very wide and lobe-shaped protruding dorsally and apically and third tooth much smaller (Figs 41, 42, 71, 74); upper valve of ovipositor enlarged and enclosing the small lower valve; third tergite sculptured in non-Afrotropical species; head often more square in dorsal view (i.e., head length longer relative to head width; Figs 47, 67); [mandible large and ventrally sinuate, with a more or less protruding small lobe (“fourth tooth”); vein 2-SR of fore wing curved basally and longer than vein 3-SR]	**4**
–	Clypeus obtuse ventrally, semicircular (Figs 12, 18, 24); upper valve of ovipositor cylindrical or depressed (*Parabobekoides*: Fig. 2; unknown of *Hovalysia* and *Neodiasta* type species); third tergite smooth; head length shorter relative to head width in dorsal view (Figs 8, 28, 55, 88, 94)	**5**
4	Vein r-m of fore wing distinctly oblique, angle with vein 2-M acute (Fig. 40); anterior tentorial pits distinctly impressed (Fig. 43); fourth to sixth metasomal tergites of ♀ largely exposed (Fig. 50); third tergite smooth (Fig. 51); Afrotropical	***Bobekoides* van Achterberg, 1998**
–	Vein r-m of fore wing vertical or slightly oblique, angle with vein 2-M about rectangular (Fig. 65); anterior tentorial pits hardly impressed (Fig. 70); fourth and following tergites largely retracted of ♀ (cf. Fig. 78); third metasomal tergite sculptured (Fig. 66); [third antennal segment nearly always distinctly widened, 1.4–2.0× wider than fourth segment (except in *H. loasensis*) and usually distinctly shorter than fourth segment: Fig. 73]; Eastern Palaearctic, Oriental	***Hylcalosia* Fischer, 1967**
5	Vein SR1 of fore wing slightly longer than vein 3-SR (Fig. 79); vein M+CU of hind wing shorter than vein 1-M (Fig. 80); pterostigma parallel-sided or narrow elliptical (Fig. 79); Neotropical	***Neodiasta* van Achterberg, gen. nov.**
–	Vein SR1 of fore wing much longer than vein 3-SR (Figs 5, 20, 26, 53); vein M+CU of hind wing at least 1.2 × as long as vein 1-M (Figs 6, 20, 26, 92); pterostigma moderately wide elliptical or triangular (Figs 5, 26, 92)	**6**
6	Vein r issued near middle of pterostigma (Fig. 53); veins r, 1-SR and 1-M of fore wing of ♂ widened (Fig. 53); precoxal sulcus nearly horizontal (Fig. 64); clypeus 2.5–3.0 × wider than high (Fig. 58); [metanotum obtuse dorsally]; Afrotropical	***Hovalysia* Granger, 1949**
–	Vein r issued behind middle of pterostigma (Figs 1, 17); veins r, 1-SR and 1-M of fore wing of ♂ slender (Fig. 20); precoxal sulcus oblique (Figs 7, 17) or absent; clypeus 2.0–2.5 × wider than high (Figs 12, 98, 121)	**7**
7	Precoxal sulcus absent (Fig. 102); in lateral view medial part of face distinctly protruding dorsally in front of antennal socket and its dorsal half nearly straight in profile (Fig. 102; Fig. 110.8 in Belokobylskij 1998); mandible straight ventrally, without fourth protuberance and hardly widened dorsally (Figs 102, 103; fig. 110.11 in Belokobylskij 1998); Holarctic	***Phasmidiasta* Wharton, 1980**
–	Precoxal sulcus present, usually wide and oblique (Figs 7, 17, 38, 125); in lateral view medial part of face normal, less protruding and its dorsal half evenly curved in profile (Figs 1, 14, 38, 125); mandible sinuate ventrally, with fourth protuberance more or less developed and widened dorsally (Figs 15, 17, 25, 32, 39, 117, 120)	**8**
8	Mandible comparatively slender, and its first tooth less protruding dorsally (Fig. 39); metanotum distinctly protruding in lateral view (Fig. 38); third antennal segment about as long as fourth segment (Fig. 34); [♂ has according to fig. 109.18 in Belokobylskij (1998) pterostigma modified, but vein r short, approx. 0.3× width of pterostigma]; Palaearctic, Afrotropical	***Bobekia* Niezabitowski, 1910**
–	Mandible robust (Figs 1, 14, 17, 125), its first tooth distinctly protruding dorsally (Figs 14, 16, 117); metanotum hardly or not protruding dorsally (Figs 1, 7, 125); third antennal segment shorter than fourth segment (Figs 10, 22, 123), but sometimes only slightly so; [♂ has modified pterostigma, but vein r medium-sized, approx. 0.7× width of pterostigma: Figs 17, 20]; Oriental, Eastern Palaearctic; genus *Separatatus* Chen & Wu, 1994	**9**
9	Base of vein 1-R1 of fore wing widened, more so in ♂♂ than in ♀♀ (Figs 1, 5, 17, 20); setose part of ovipositor sheath 1.5–1.6× longer than metasoma and 0.6–0.7× as long as fore wing (Fig. 1); upper valve of ovipositor flattened dorsally; hind femur 4.4–5.3× longer than wide (Figs 1, 17); propodeal areola reduced anteriorly (Figs 9, 21)	**subgenus Parabobekoides van Achterberg & Tan, subg. nov.**
–	Base of vein 1-R1 of fore wing narrow (♀: Fig. 115; ♂unknown); setose part of ovipositor sheath 0.8–1.0× longer than metasoma and 0.3–0.4 × as long as fore wing (Fig. 125); upper valve of ovipositor (at least in type species) normal, convex dorsally; hind femur 2.7–3.5× longer than wide (Fig. 119); propodeal areola complete anteriorly (Fig. 116), except in *S. malaysiae*	**subgenus Separatatus Chen & Wu, 1994**

**Notes.***Phasmalysia* Tobias, 1971 (type species: *Phasmalysia zinovjevi* Tobias, 1971, from S. Russia [examined]) might belong to the *Bobekia* group, but it is excluded here because of the uniquely shaped mandible (first tooth extremely enlarged and lobe-shaped, with curved carina present on first and third tooth and mandible consisting mainly of two large lobes (if viewed with full sight on the first tooth) because of the deep medio-apical incision). The type species has the third antennal segment slender, and the second tergite only superficially sculptured. The Nearctic *P. borealis* Wharton, 1980, has the second tergite more sculptured, but is also characterized by an aberrantly shaped mandible.

## Taxonomy

### Separatatus

Taxon classificationAnimaliaHymenopteraBraconidae

Chen & Wu, 1994

54028A37-ED54-5189-BFC0-909B62331097

Figs 1–4 , 5–16 , 17–19 , 20–25 , 115–126

Separatatus Chen & Wu, 1994: 132; Zhu et al. 2017: 69–72; Yao et al. 2018a: 187–188. Type species (by monotypy): Separatatus carinatus Chen & Wu, 1994 [holotype (FAFU) examined].
Phasmidiasta sensu Fischer 2006: 628–631 (p.p.).
Hovalysia sensu Wharton 2002: 79 (figs 124–127).
Bobekoides sensu Zheng et al. 2013: 143–146 (p.p.).

#### Notes.

A small Oriental and East Palaearctic genus in terms of species richness; hosts are unknown for all species. Species of *Separatatus* can be identified with the key by Yao et al. (2018a), and those of *Parabobekoides* with the key below.

**Figures 1–4.**
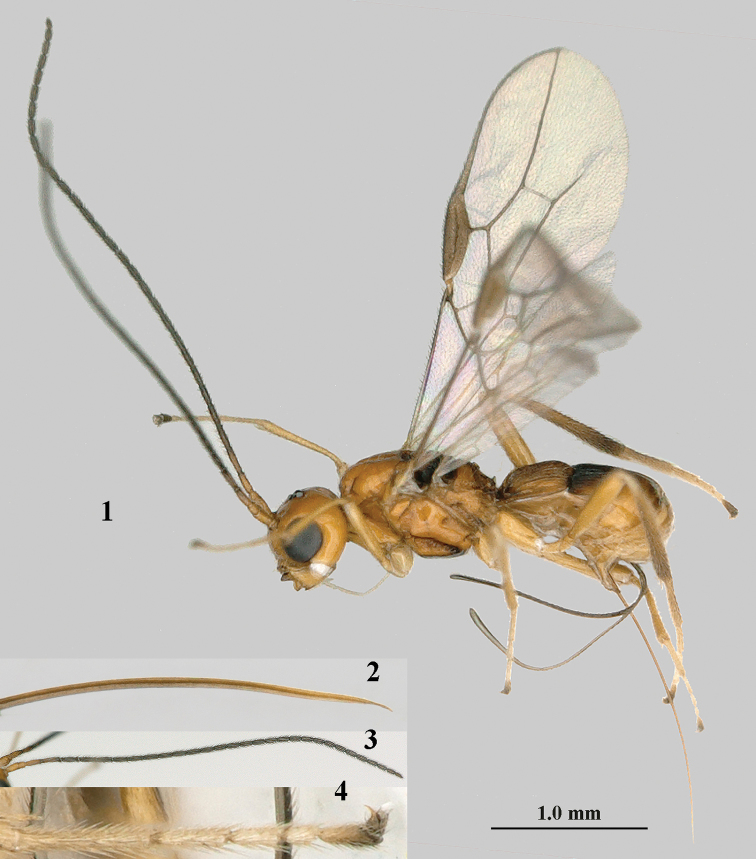
*Separatatus yinshani* Zhang & van Achterberg, sp. nov., ♀, holotype **1** habitus, lateral aspect **2** apex of ovipositor, lateral aspect **3** antenna **4** middle tarsus and outer claw, lateral aspect.

### Subgenus Parabobekoides

Taxon classificationAnimaliaHymenopteraBraconidae

van Achterberg & Tan subg. nov.

BF803240-BC1B-59A3-8410-FEB55B6BA091

http://zoobank.org/79C99F4A-C69C-4B45-97BF-FC1C800C3412

#### Type species.

Separatatus (Parabobekoides) yinshani Zhang & van Achterberg, sp. nov. Gender: masculine.

#### Diagnosis.

**Figures 5–16.**
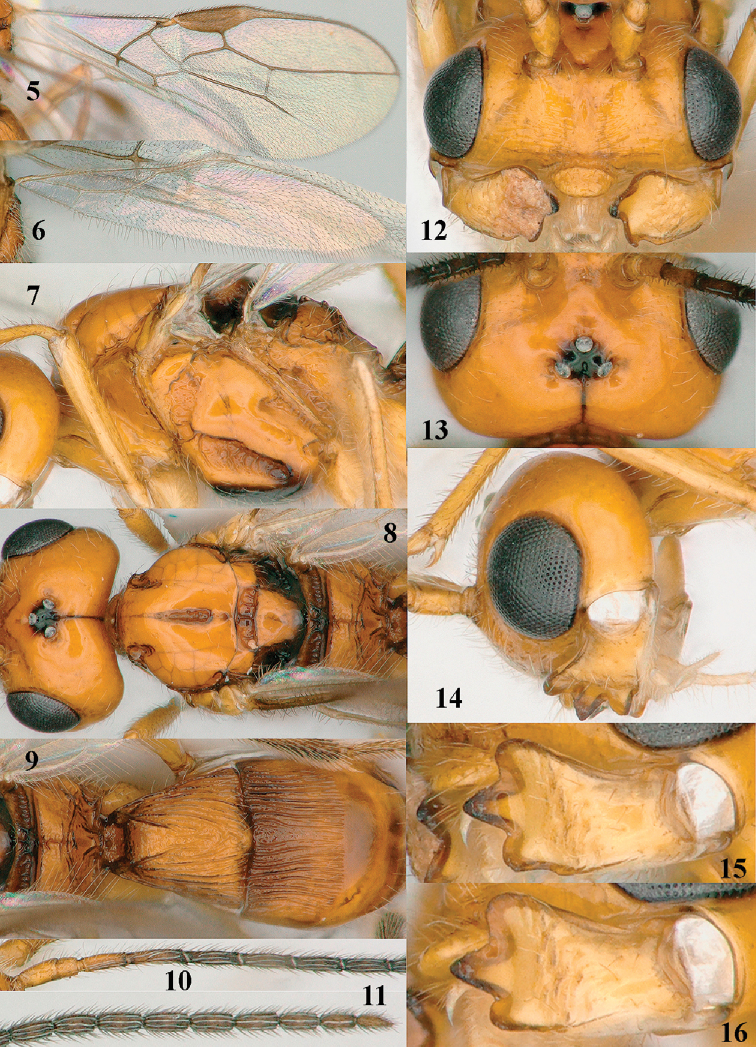
*Separatatus yinshani* Zhang & van Achterberg sp. nov., ♀, holotype **5** fore wing **6** hind wing **7** mesosoma, lateral aspect **8** head and mesosoma, dorsal aspect **9** propodeum, first–third metasomal tergites, dorsal aspect **10** basal antennal segments **11** apical antennal segments **12** head, anterior aspect **13** head, dorsal aspect **14** head, lateral aspect **15** mandible, full view of third tooth **16** mandible, full view of first tooth.

**Figures 17–19.**
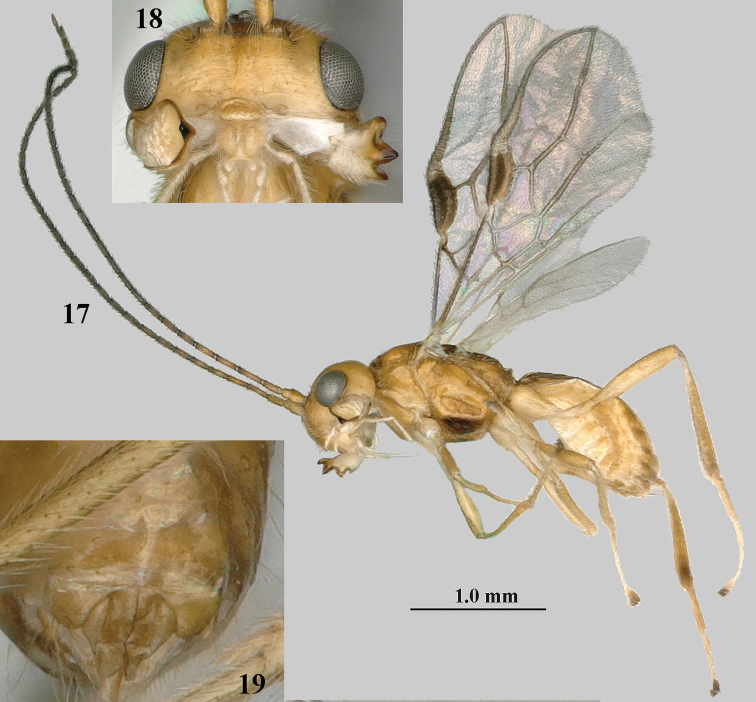
*Separatatus yinshani* Zhang & van Achterberg sp. nov., ♂, paratype **17** habitus, lateral aspect **18** head, anterior aspect **19** genitalia, ventral aspect.

**Figures 20–25.**
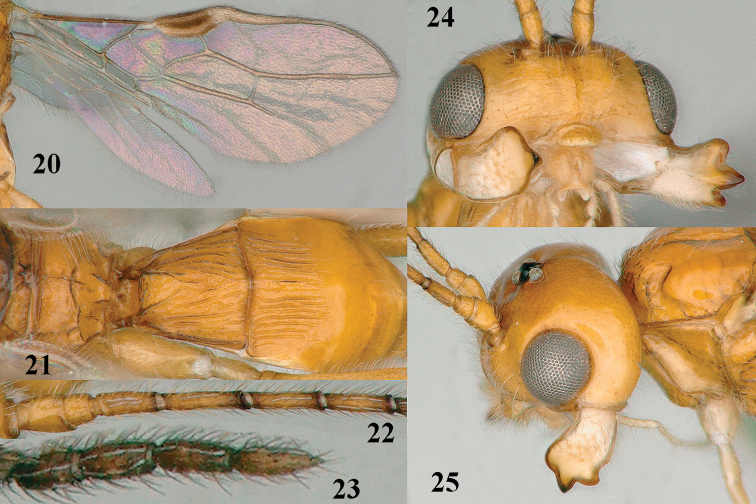
*Separatatus yinshani* Zhang & van Achterberg sp. nov., ♂, paratype **20** wings **21** propodeum, first–third metasomal tergites, dorsal aspect **22** basal antennal segments **23** apical antennal segments **24** head, anterior aspect **25** head, lateral aspect.

Propodeal areola reduced anteriorly (Figs 8, 9, 21); setose part of ovipositor sheath distinctly longer than metasoma and 0.6–0.7× as long as fore wing (Fig. 1); upper valve of ovipositor flattened apically (Fig. 2). Superficially, the new subgenus is very similar to *Bobekoides* van Achterberg and shares the derived shape of the upper valve of the ovipositor, but differs by the semicircular clypeus (Fig. 12; acute and triangular in *Bobekoides*), vein r-m of the fore wing nearly straight and angle with vein 2-M about 90° (Figs 1, 5, 17, 20; distinctly inclivous and angle distinctly less than 90° in *Bobekoides*), the transverse head in dorsal view (Fig. 8; more square in *Bobekoides*), the basally widened and more or less differentiated vein 1-R1 (narrow and not differentiated in *Bobekoides*), the distinct sexual dimorphism of the fore wing venation (Fig. 20; absent in *Bobekoides*), the posteriorly wide propodeal areola (Fig. 21; narrow in *Bobekoides*) and the mandible less massively enlarged dorsally , its dorsal tooth somewhat wider than second (= middle) tooth (Fig. 15; strongly enlarged dorsally, dorsal tooth much wider than second tooth in *Bobekoides*).

#### Distribution.

China (Hubei, Shaanxi).

#### Etymology.

“Para” is Greek for “beside, near, by” and the generic name *Bobekoides*, because it is similar to this genus.

##### Key to species of subgenus Parabobekoides, subg. nov. 

**Table:** 

1	Antenna of ♀ with ca 47 segments and 1.7× longer than fore wing; face transversely rugose laterally; mesoscutum largely blackish brown; striae of second tergite partly distinctly oblique	**S. (P.) sinicus (Zheng, Chen & Yang, 2013)**
_	Antenna of ♀ with 31–33 segments and 1.3–1.4× longer than fore wing (of ♂ up to 1.5×); face smooth laterally, remainder largely superficially rugulose (Figs 12, 18); mesoscutum yellowish brown; striae of second tergite largely longitudinal or nearly so (Figs 9, 21)	**S. (P.) yinshani Zhang & van Achterberg, sp. nov.**

##### Discussion

Zheng et al. (2013) reported *Bobekoides sinicus* Zheng, Chen & Yang, 2013 from Central China (Hubei). This was the first time that a species of *Bobekoides* van Achterberg, 1998 was reported from outside Africa. Zhu et al. (2017) included this species in the genus *Separatatus* Chen & Wu, 1994, because it has an obtuse clypeus as in the type species of *Separatatus*. In *Bobekoides*, the clypeus is acute and triangular (Fig. 43).

In 2017 a series of a similar species was collected at Luonan (Qinling Mountains, Shaanxi, NW China) in which males have the venation modified (Figs 17, 20) in comparison to females. The venation is also modified in the male of the type species of *Hovalysia* Granger, 1949, known only from the Afrotropical region, of which the female is unknown. Wharton (2002) reported the occurrence of *Hovalysia* in China (Taiwan), but the lack of females did not allow for a proper inclusion in the key by Zhu et al. (2017), and it was left out pending the availability female specimens. Fischer (1999) described *Hovalysia cruciata* from South Africa based on one female specimen, but he did not indicate the shape of the ovipositor. The series from Luonan include females with normal (= slender) veins 3-SR and 2-M, vein 2-SR about as long as vein 3-SR, with a modified upper valve of the ovipositor, and vein r 0.6× width of pterostigma (Fig. 5). The males have the basal part of vein 1-R1 wider than in females, veins 3-SR and 2-M widened, vein 2-SR distinctly shorter than vein 3-SR, and vein r about 0.7× as wide as the pterostigma (Fig. 20). Inclusion in *Hovalysia* is a possibility, but is problematic because the Chinese specimens have the first mandibular tooth wide, lobe-shaped, and strongly protuberant both dorsally and apically (rectangular, not protruding apically and hardly so dorsally in *Hovalysia*); the males have a different pattern of widened veins (e.g., veins 1-SR, 1-M, and r are widened in *Hovalysia* and slender in Chinese males); and vein CU1b of the fore wing is shorter than vein 3-CU1 (as long as vein 3-CU1 in *Hovalysia*).

### Separatatus (Parabobekoides) yinshani

Taxon classificationAnimaliaHymenopteraBraconidae

Zhang & van Achterberg sp. nov.

793863F4-E8EF-5255-A69B-BA90505E7A25

http://zoobank.org/AB358A9B-BE34-418B-A823-6A3320D4A49C

Figs 1–4 , 5–16 , 17–19 , 20–25

#### Type material.

***Holotype***: ♀ (NWUX), “NW. China: Shaanxi, Luonan, Shangluo, 34.03N, 110.10E, 9.vii.–9.ix.2017, alt. 1006 m, B[lack] Mal[aise] trap, Tan Jiangli, NWUX”. ***Paratypes***: 5 ♂ + 1 ♀ (NWUX, RMNH), same data.

#### Diagnosis.

Antenna of ♀ with 31–33 segments and 1.3–1.4× longer than fore wing; face smooth laterally and remainder largely superficially rugulose (Figs 12, 18, 24); mesoscutum yellowish brown; vein r of fore wing 0.3 × as long as vein 3-SR and 0.5–0.6 (♀) – 0.7 (♂) × width of pterostigma (Figs 1, 5, 17, 20); striae of second tergite largely longitudinal or nearly so (Figs 9, 21); setose part of ovipositor sheath approx. 0.7× as long as fore wing and nearly twice as long as hind tibia (Fig. 1).

#### Description.

Holotype, ♀, length of body 2.6 mm, of fore wing 2.8 mm.

***Head***: Moderately transverse and shiny, slightly concave posteriorly (Fig. 8), width of head 1.8× its lateral length; antenna with 31 segments and 1.4× longer than fore wing, segments with long bristly setae, third segment 0.8× as long as fourth segment and 1.3× wider than fourth segment in lateral view, length of third, fourth and penultimate segments 2.4, 4.0 and 2.8× their width, respectively (Figs 10, 11); length of maxillary palp 1.3× height of head; eye in dorsal view 1.5× as long as temple (Fig. 8); frons depressed in front of anterior ocellus and with shallow reversed V-shaped depression anteriorly (Fig. 13); vertex convex and very sparsely setose; OOL: diameter of ocellus: POL = 15:4:5; face 2.1× wider than high, largely smooth laterally and dorsally, superficially rugulose medially but with longitudinal convex median area smooth (Fig. 12); clypeus 2.2× wider than high, protruding, semicircular and nearly truncate medio-ventrally (Fig. 12); malar space virtually absent; mandible moderately widened dorsally and ventrally sinuate, dorsal tooth large and lobe-shaped, similar to ventral tooth and with minute ventral protuberance, middle tooth curved and robust; medial length of mandible 1.5× its maximum width (Figs 14–16).

***Mesosoma***: Length of mesosoma 1.5× its height; mesoscutum with lateral carina in front of tegulum distinct (Fig. 7); pronotal sides smooth except for oblique carina anteriorly; epicnemial area widely depressed and partly crenulate (Fig. 7); precoxal sulcus very wide, oblique, coarsely crenulate, up to anterior depression but absent posteriorly (except short depression above middle coxa; Fig. 7); remainder of mesopleuron smooth and largely glabrous; pleural sulcus narrowly crenulate; episternal scrobe medium-sized and oblique; metapleuron largely smooth but with some coarse carinae posteriorly, with long setae and rather small pit anteriorly; pronope medium-sized compared to length of pronotum and nearly round (Fig. 8); notauli crenulate and wide, but only anteriorly impressed on disc; medio-posterior depression of mesoscutum long and deep, finely crenulate and up to level of notauli (Fig. 8); mesoscutum strongly shiny and smooth, with some setae anteriorly and posteriorly of notauli; scutellar sulcus deep and wide, with 5 carinae and 3× wider than its maximum length; scutellum rather convex and smooth, sparsely setose (Fig. 8; metanotum hardly protruding medio-posteriorly and only anterior half with median carina; medio-longitudinal carina of propodeum medium-sized, connected to (partly double) curved carina and areola incomplete, only posteriorly with pair of curved carinae and laterally crenulate, remainder largely smooth (Figs 8, 9).

***Wings*** (Figs 1, 5, 6): Pterostigma elliptical, rather swollen, apically differentiated from widened basal part of 1-R1; vein r 0.6× width of pterostigma; r: 3-SR:SR1 = 5:19:52; SR1 straight and 2-SR curved posteriorly; cu-a subinterstitial, short; 3-CU1 much longer than CU1b; 2-SR: 3-SR: r-m = 19:19:11; m-cu postfurcal, strongly converging to 1-M posteriorly; first subdiscal cell 2.7× as long as wide; M+CU1 largely sclerotized. Hind wing: M+CU: 1-M: 1r-m = 23:17:10; m-cu faintly indicated.

***Legs***: Hind coxa smooth; tarsal claws rather robust and shorter than arolium (Fig. 4); length of femur, tibia and basitarsus of hind leg 5.3, 11.2 and 4.4 × their width, respectively; hind leg rather conspicuously setose.

***Metasoma***: Length of first tergite equal to its apical width, its surface largely coarsely longitudinally striate (but striae partly converging posteriorly), its dorsal carinae widely separated (Fig. 9); dorsope distinct, medium-sized (Fig. 9); second tergite entirely coarsely longitudinally striate; third tergite smooth and in lateral view distinctly convex (Fig. 1); setose part of ovipositor sheath with rather short and dense setae, 0.61× as long as fore wing (total visible sheath (including glabrous band-shaped part) 0.68×), 1.5× metasoma, 3.2× first tergite and 1.7× as long as hind tibia; hypopygium acute apically and weakly sclerotized.

***Colour***: Brownish yellow; mandible, palpi, tegulum, humeral plate and legs (but hind tibia (except basally) and tarsus infuscate) pale yellowish or ivory; antenna (except 3 basal segments), mesosternum largely, scutellum laterally and posteriorly and ovipositor sheath dark brown; pterostigma (except pale yellowish apex) and most veins brown; second and third tergites slightly darkened; wing membrane subhyaline.

***Variation***: The wing venation of males show distinct sexual dimorphism (Figs 17, 20), the pterostigma is enlarged and apically distinctly differentiated from the strongly widened basal part of vein 1-R1. Additionally, veins 3-SR, 2-M, and SR1 of fore wing are widened. The body length of females is 2.5–3.0 mm and, of males, 2.6–2.8 mm; the length of the fore wing of females is 2.8–3.0 mm and, of males, 2.7–3.0 mm; the antennal segments of females is 31(1), 33(1) and, of males, 29(1), 31(2), 32(1), and 33(1); the antenna is 1.3–1.5× as long as the fore wing. The setose part of the ovipositor sheath is 0.61–0.63× as long as the fore wing. The mesosternum is brownish yellow or largely dark brown, and up to basal third of the antenna may be brownish yellow or brown.

#### Etymology.

Named after the father of one of the co-authors (RNZ) in recognition of his support for many years.

### Bobekia

Taxon classificationAnimaliaHymenopteraBraconidae

Niezabitowski, 1910

3FE59441-7597-57C5-8E2A-17CB42106817

Figs 26–39

Bobekia Niezabitowski, 1910: 102; Fischer 1971: 137–139 (as synonym of Symphanes Foerster, 1863); Shenefelt 1974: 1020 (id.); Wharton 1980: 68 (id.); van Achterberg 1998: 106 (as valid genus). Type species: Bobekia montana Niezabitowski, 1910, designated by Shenefelt (1974) (= Alysia striolata Thomson, 1895; synonymized by Fischer (1974)) [holotype (PAN) examined].
Neosymphanes Belokobylskij, 1998: 294 (as subgenus of Symphanes Foerster, 1863). Type species (by original designation): Alysia striolata Thomson, 1895 [holotype (ZIL) examined]. Syn. nov.

#### Notes.

A small genus with species from the Palaearctic and Afrotropical regions of which the type species and only named Palaearctic species has been reared from Agromyzidae (Yu et al. 2016). One of us (CvA) has seen Afrotropical specimens (RMNH) reared from mining Muscidae (*Atherigona* sp.). Unfortunately, host specimens were not retained and no additional data exists on how the specimens were reared.

As indicated by van Achterberg (1998), the genus *Symphanes* Foerster, 1863, is morphologically heterogeneous and does not belong to the *Bobekia*-group. Also, *Bobekia* is a separate genus from *Symphanes*. *Symphanes* is excluded because of the absence of a distinct dorsope in the first metasomal tergite (distinctly developed in *Bobekia*), tarsal claws angulate (evenly curved in *Bobekia*), first subdiscal cell of fore wing narrowly open (closed in *Bobekia*), and third antennal segment slightly longer than fourth segment (slightly shorter in *Bobekia*). *Neosymphanes* is a junior synonym of *Bobekia* because they share the same type species, *Bobekia montana* Niezabitowski, 1910, which was synonymized with *Alysia striolata* Thomson, 1895 by Fischer (1974). The differences between the types of *B. montana* and *A. striolata* are minimal: the ovipositor sheath is more retracted in *A. striolata* and vein 3-CU1 of the fore wing is distinctly longer than CU1b in *B. montana* (Fig. 36) and about equal in *A. striolata*.

**Figures 26–39.**
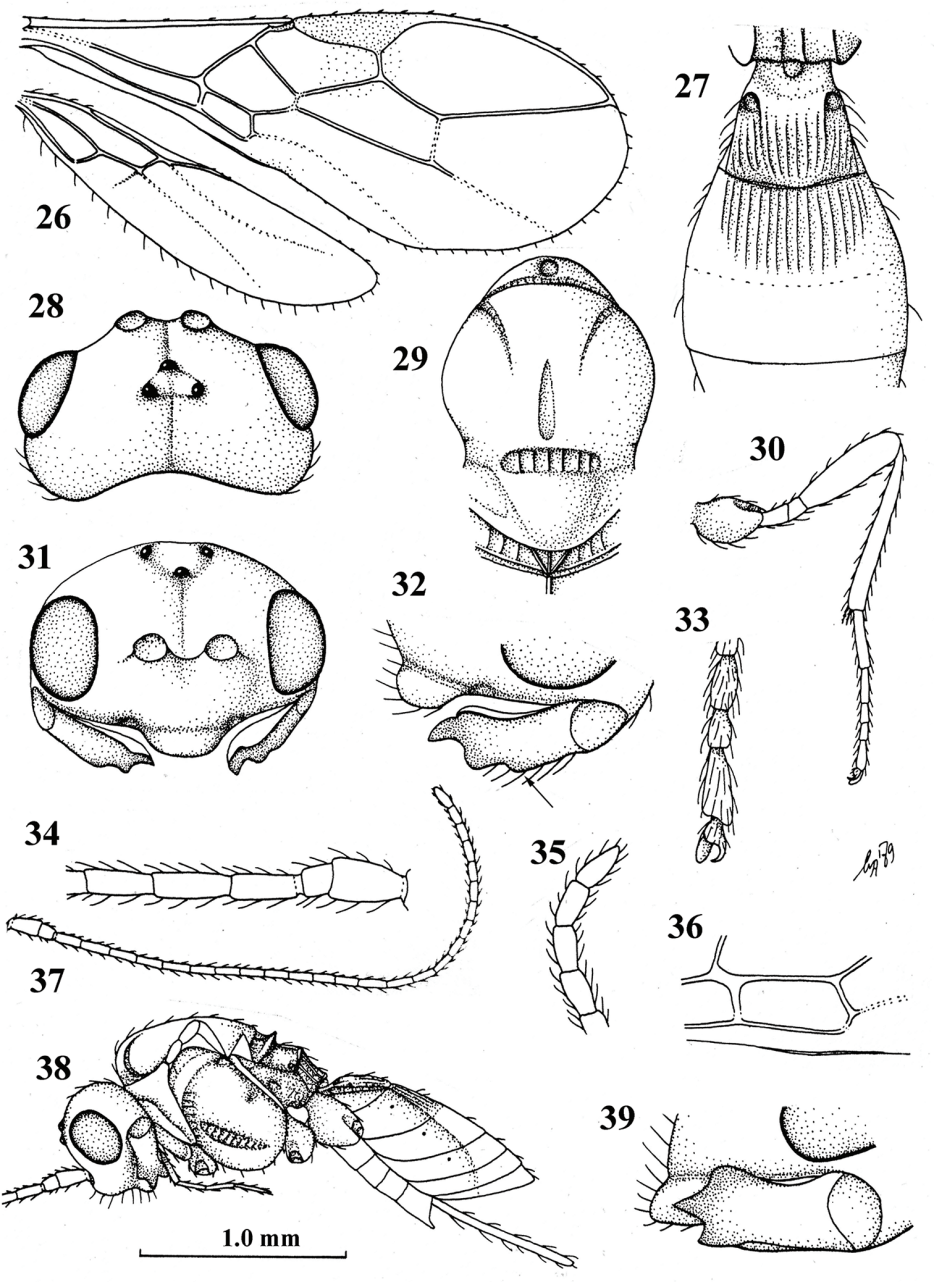
*Bobekia montana* Niezabitowski, ♀, holotype **26** wings **27** first–third metasomal tergites, dorsal aspect **28** head, dorsal aspect **29** mesosoma, dorsal aspect **30** hind leg **31** head, anterior aspect **32** mandible, full view of third tooth (fourth tooth arrowed) **33** outer hind claw, lateral aspect **34** basal antennal segments **35** apical antennal segment **36** detail of first subdiscal cell of fore wing **37** antenna **38** habitus, lateral aspect **39** mandible, full view of first tooth.

### Bobekoides

Taxon classificationAnimaliaHymenopteraBraconidae

van Achterberg, 1998

91E07E48-9D44-537A-81E7-FBA090378270

Figs 40–52

Bobekoides van Achterberg, 1998: 105; Zheng et al. 2013: 143. Type species (by original designation): Bobekoides fulvus van Achterberg, 1998 [holotype (ZIL) examined].

#### Notes.

A genus with a few species in the Afrotropical region. The biology is unknown. See van Achterberg (1998) for a key to species; see *Separatatus* for the Chinese species.

**Figures 40–52.**
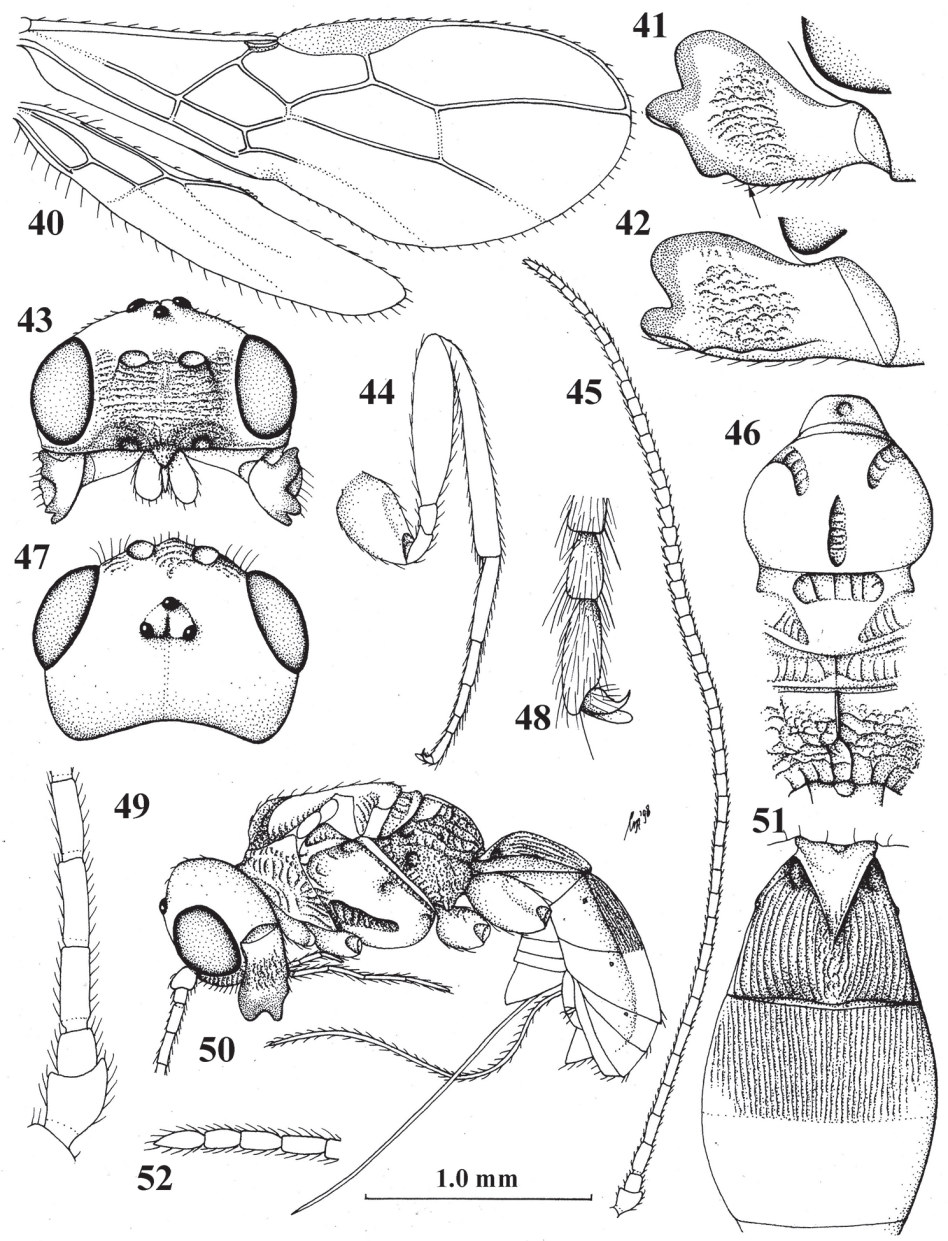
*Bobekoides fulvus* van Achterberg, ♀, holotype **40** wings **41** mandible, full view of third tooth (fourth tooth arrowed) **42** mandible, full view of first tooth **43** head, anterior aspect **44** hind leg **45** antenna **46** mesosoma, dorsal aspect **47** head, dorsal aspect **48** outer hind claw, lateral aspect **49** basal antennal segments **50** habitus, lateral aspect **51** first–third metasomal tergites, dorsal aspect **52** apical antennal segments.

### Hovalysia

Taxon classificationAnimaliaHymenopteraBraconidae

Granger, 1949

2DED895F-C0BC-51E3-8B14-BC77BDFFE579

Figs 53–64

Hovalysia Granger, 1949: 400; Shenefelt 1974: 992; Fischer 1993: 609–611, 1999: 9–10. Type species (by monotypy); Hovalysia seyrigi Granger, 1949 [holotype (MNHN) examined].

#### Notes.

**Figures 53–64.**
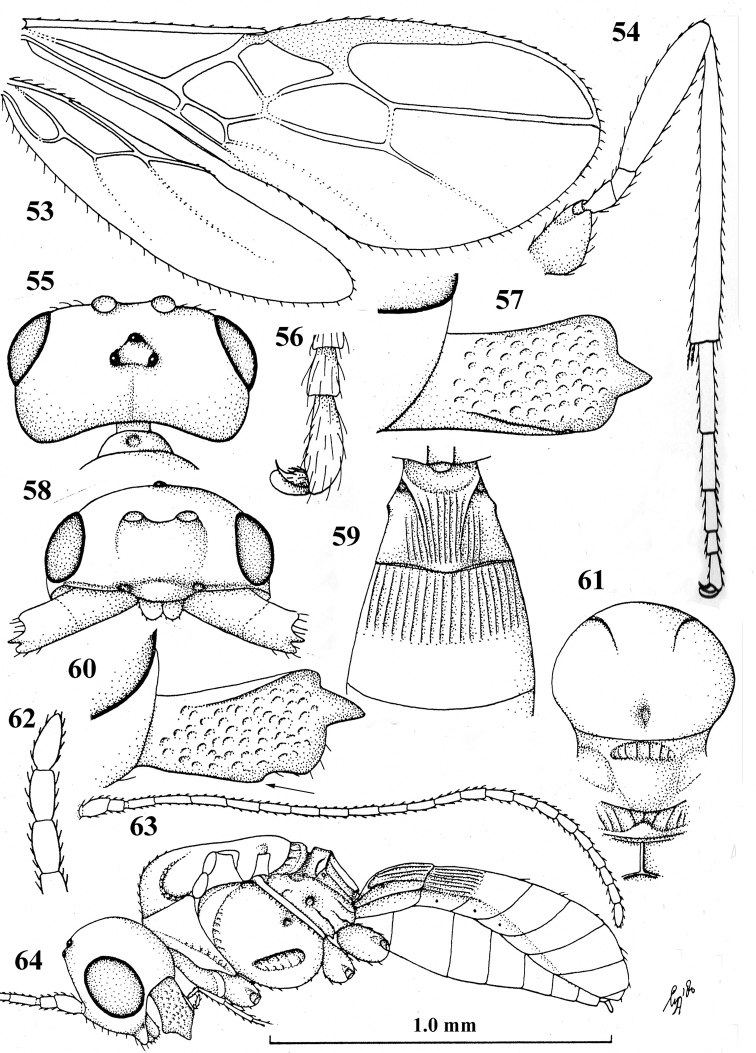
*Hovalysia seyrigi* Granger, ♂, holotype **53** wings **54** hind leg **55** head, dorsal aspect **56** outer hind claw, lateral aspect **57** mandible, full view of first tooth **58** head, anterior aspect **59** first–third metasomal tergites, dorsal aspect **60** mandible, full view of third tooth (fourth tooth arrowed) **61** mesosoma, dorsal aspect **62** apical antennal segments **63** antenna **64** habitus, lateral aspect.

The main characters are the medial position of vein r at the pterostigma and the aberrant venation of the fore wing in males (Fig. 53), which is unknown in other genera. Only two Afrotropical species are known. For their identification, see the key by Fischer (1999). So far, no males are known in China with similar venation, but Chinese male *Separatatus* with aberrant venation (Figs 17, 20) are known. Therefore, Wharton’s (2002) reference to Taiwanese specimens with widened veins probably refers to a species of *Separatatus*. The biology is unknown.

### Hylcalosia

Taxon classificationAnimaliaHymenopteraBraconidae

Fischer, 1967

0C6DCFC8-B4EC-5B9B-96CC-2F3A0731F4C7

Figs 65–78

Holcalysia Cameron 1910: 6; Shenefelt 1974: 993. Type species (by monotypy): Holcalysia ruficeps Cameron, 1910 [holotype (ZMB) examined].
Hylcalosia Fischer, 1967: 125 (replacement name for Holcalysia Cameron, 1910 (not Cameron 1905), 2008: 718–722; Shenefelt 1974: 993; van Achterberg 1983: 81; Belokobylskij 1992: 143, 1998: 297, 2015: 530; Chen and Wu 1994: 85; Papp 1994: 139–142; Wharton 2002: 23; Zheng et al. 2012: 454; Zhu et al. 2017: 63–64, 2018: 548; Yao et al. 2019: 4. Type species (by monotype): Holcalysia ruficeps Cameron, 1910 [holotype (ZMB) examined].

#### Notes.

A rather small Palaearctic and Oriental genus, of which the biology is unknown. Wharton (2002) included it in his generic key for Australia, suggesting its occurrence in the Australasian region. *Hylcalosia* species show several apomorphic character states within the *Bobekia*-group, as expressed by the shape of the clypeus, mandible (especially in the type species; Fig. 74), and metasoma (more or less carapace-like; Fig. 78). Identification keys to species were given by Zhu et al. (2018) and Yao et al. (2019).

**Figures 65–78.**
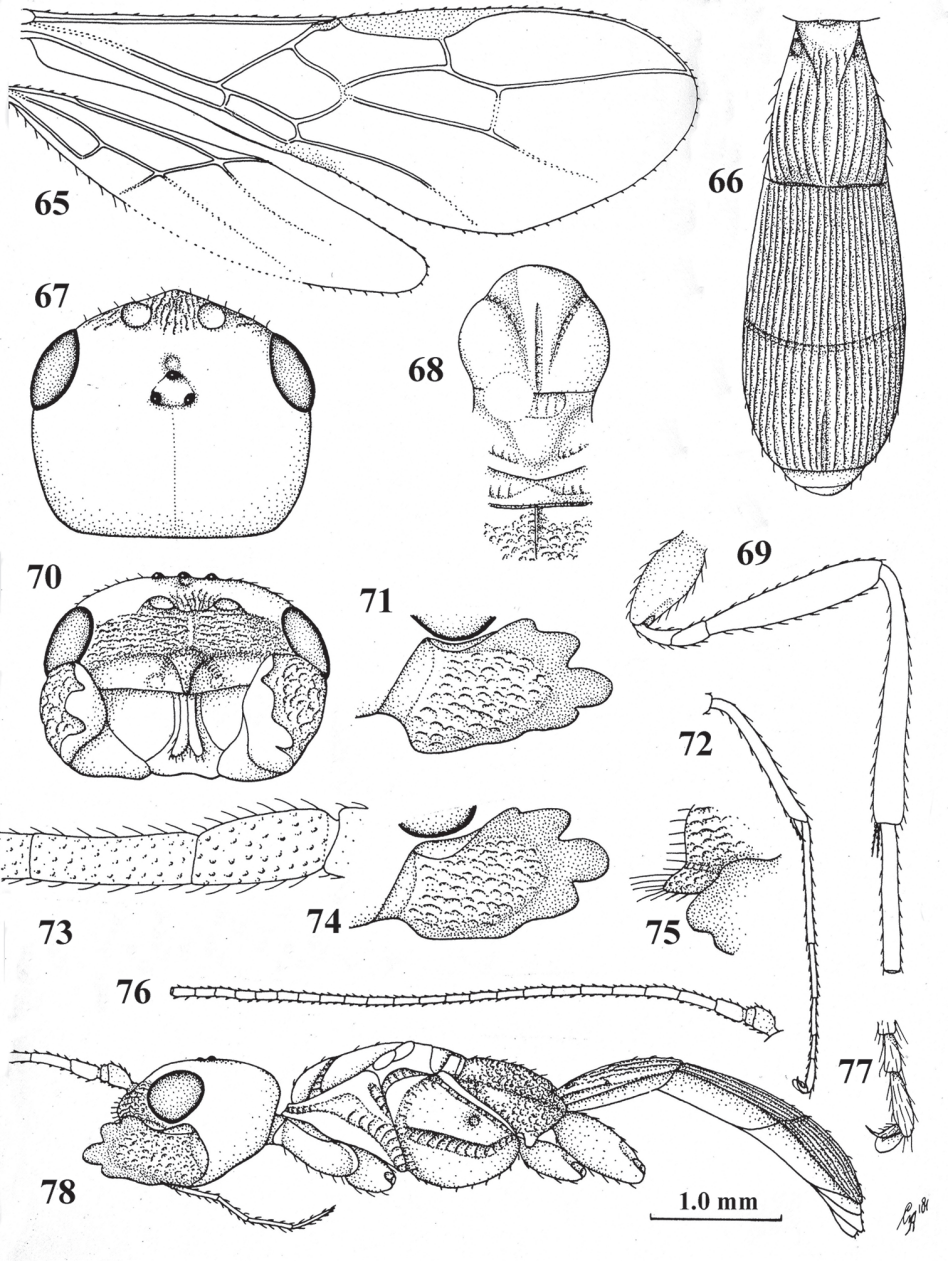
*Hylcalosia ruficeps* (Cameron), ♂, holotype **65** wings **66** first–third metasomal tergites, dorsal aspect **67** head, dorsal aspect **68** mesosoma, dorsal aspect **69** hind leg **70** head, anterior aspect **71** mandible, full view of first tooth **72** fore tibia and tarsus **73** basal antennal segments **74** mandible, full view of third and fourth teeth **75** clypeus lateral aspect **76** antenna **77** outer fore claw, lateral aspect **78** habitus, lateral aspect.

### Neodiasta

Taxon classificationAnimaliaHymenopteraBraconidae

van Achterberg gen. nov.

EE910EFB-E601-5A80-9E7C-8434FBD88734

http://zoobank.org/10A95EB9-7224-4CF3-A65A-A96C9E69A499

Figs 79–91

#### Type species.

*Phasmidiasta ecuadorensis* Fischer, 2006.

#### Diagnosis.

**Figures 79–91.**
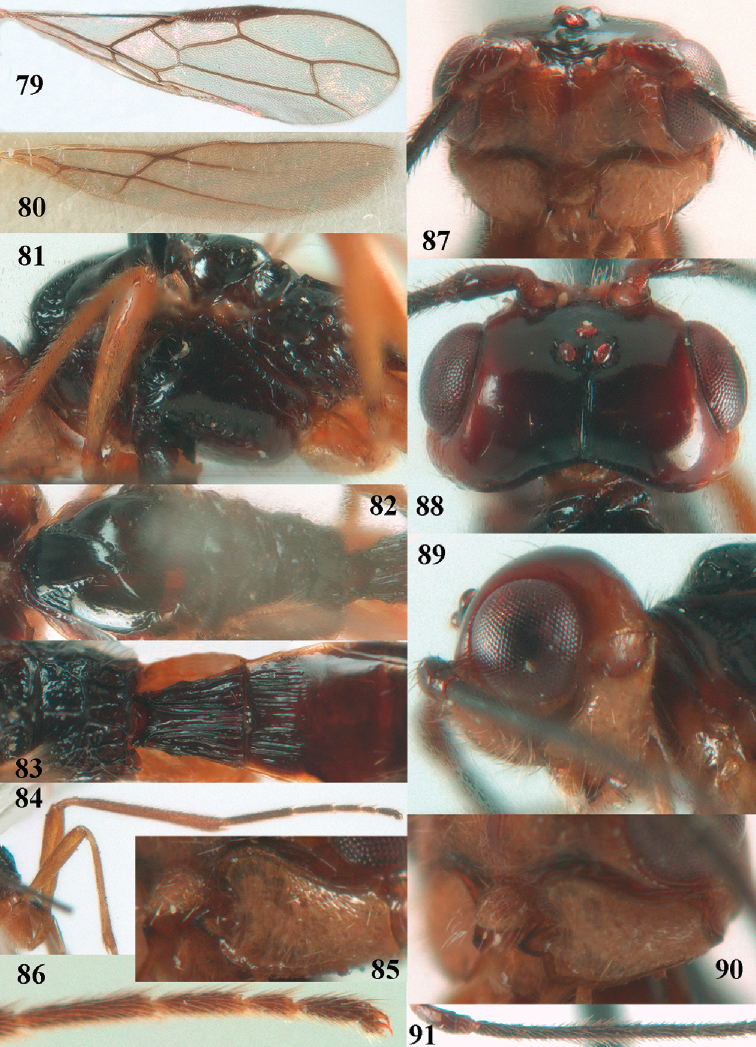
*Neodiasta ecuadorensis* (Fischer), ♂, holotype **79** fore wing **80** hind wing **81** mesosoma, lateral aspect **82** mesosoma, dorsal aspect **83** propodeum, first–third metasomal tergites, dorsal aspect **84** hind leg **85** mandible, full view of first tooth **86** outer hind claw **87** head, anterior aspect **88** head, dorsal aspect **89** head, lateral aspect **90** mandible, full view of third tooth **91** basal antennal segments.

Third antennal segment shorter than fourth segment and slender (Fig. 91); mandible strongly widened apically, with minute ventral lobe and no oblique ventral carina, with 3 large teeth, middle tooth much smaller than upper tooth, upper tooth without dorso-apical protuberance, ventral margin straight but near third lobe-shaped tooth with minute lobe (Figs 85, 90); clypeus obtuse ventrally, semicircular (Figs 85, 87, 90); face normally convex and not protruding medially (Figs 87, 89); pronope deep and medium-sized (Fig. 88); precoxal sulcus wide and coarsely crenulate medially; vein 2-SR of fore wing straight posteriorly (Fig. 79); vein r of fore wing issued behind medially from pterostigma and pterostigma parallel-sided to narrow elliptical (Fig. 79); vein CU1b of fore wing distinctly shorter than vein 3-CU1 and vein CU1a distinctly below level of vein CU1 (Fig. 79); first subdiscal cell of fore wing closed distally and moderately wide (Fig. 79); vein M+CU of hind wing distinctly shorter than vein 1-M (Fig. 80); first metasomal tergite with distinct dorsope; second tergite distinctly striate basally (Fig. 83) and third tergite smooth; shape of ovipositor and length of ovipositor sheath unknown (only ♂ known).

#### Distribution.

Neotropical (one species).

#### Notes.

The biology of the only known specimen (the male holotype from Ecuador) is unknown. The types species does not fit in *Phasmidiasta* because the precoxal sulcus is present and coarsely crenulate (absent in *Phasmidiasta*), the face is medially not protruding (distinctly protruding in *Phasmidiasta*), vein SR1 of the fore wing about as long as vein 3-SR (about 4× as long in *Phasmidiasta*), vein M+CU of the hind wing is distinctly shorter than vein 1-M (longer than vein 1-M in *Phasmidiasta*), mandible without oblique carina connected to third tooth (present in *Phasmidiasta*), and the pterostigma is parallel-sided to narrowly elliptical (moderately widely elliptical to triangular in *Phasmidiasta*).

#### Etymology.

Name derived from a combination of “neo” (Greek for “new”) and the generic name *Phasmidiasta*, because it occurs in the Neotropical region and was formerly included in *Phasmidiasta*. Gender: feminine.

### Neodiasta ecuadorensis

Taxon classificationAnimaliaHymenopteraBraconidae

(Fischer, 2006) comb. nov.

E020C736-65CD-57DE-B7E3-86351167D098

Figs 79–91

Phasmidiasta ecuadorensis Fischer, 2006: 628–629.

#### Type material.

***Holotype***: ♂ (BZL), “Ecuador: Tungurahua prov., Banos, 14.ii.2002, 1500 m, M. Halada”, “♂ ***Holotype***: *Phasmidiasta ecuadorensis* sp. nov., M. Fischer, det. 2005”.

#### Diagnosis.

See genus diagnosis.

#### Description.

Holotype, ♂, length of body 4.3 mm, of fore wing 4.3 mm.

***Head***: Head moderately transverse and shiny, concave posteriorly (Fig. 88), width of head 1.8× its lateral length; antenna incomplete, 27 segments remaining and segments with long bristly setae, third segment 0.8× as long as fourth segment and 1.2× wider than fourth segment in lateral view, length of third and fourth segments 2.7 and 4.2× their width, respectively (Fig. 91); length of maxillary palp 1.8× height of head; eye in dorsal view 1.6× as long as temple (Fig. 88); frons largely flat in front of anterior ocellus and only behind antennal sockets with narrow depression (Fig. 88); vertex convex and very sparsely setose; OOL: diameter of ocellus: POL= 9:2:2; face 2.4 × wider than high, largely striate laterally, largely smooth medio-dorsally, moderately convex and with longitudinal convex median area (Fig. 87); clypeus 1.3× wider than high, protruding, semicircular and nearly truncate medio-ventrally (Fig. 87); malar space virtually absent; mandible strongly widened dorsally and ventrally straight, but near third lobe-shaped tooth with minute lobe, dorsal tooth large and lobe-shaped, larger than similar ventral tooth, middle (= second) tooth curved, small compared to first tooth and robust; medial length of mandible 1.6× its maximum width (Figs 85, 90).

***Mesosoma***: Length of mesosoma 1.6× its height; mesoscutum with lateral carina in front of tegulae distinct and crenulate; pronotal sides shiny and smooth but oblique groove crenulate anteriorly and sparsely crenulate posteriorly; epicnemial area depressed anteriorly and partly crenulate (Fig. 81); precoxal sulcus very wide, oblique, coarsely crenulate, but posterior 0.3 absent (except short depression above middle coxa; Fig. 81); remainder of mesopleuron smooth and largely glabrous except ventrally; pleural sulcus finely crenulate; episternal scrobe medium-sized and oblique; metapleuron largely smooth but with some rugae medially, with some long setae and deep pit anteriorly; mesosternal sulcus finely crenulate; pronope medium-sized (compared to length of pronotum in dorsal view), deep and nearly round (Fig. 88); notauli distinctly crenulate and wide, but posteriorly narrow and nearly smooth; medio-posterior depression of mesoscutum long and deep, smooth and up to level of notauli (Fig. 82); mesoscutum strongly shiny and smooth, largely glabrous; scutellar sulcus deep and wide, with one carina, narrowed medially and 2.4× wider than its maximum length; scutellar disc weakly convex (but posteriorly rather bulging), largely glabrous and smooth (Fig. 81); metanotum hardly protruding and only anterior half with median carina; medio-longitudinal carina of propodeum coarse and only on anterior face of propodeum, connected to complete parallel-sided areola, posterior face smooth between carinae and dorsally crenulate-rugose except smooth anterior area (Figs 81–83).

***Wings*** (Figs 79, 80). Pterostigma very narrow elliptical (nearly parallel-sided), apically hardly differentiated from 1-R1 and vein r issued slightly behind middle of pterostigma; vein r 0.8× width of pterostigma; r:3-SR:SR1 = 5:31:31; SR1 and 2-SR straight; cu-a just postfurcal; 3-CU1 much longer than CU1b; 2-SR:3-SR:r-m = 25:31:14; m-cu far postfurcal, converging to 1-M posteriorly; first subdiscal cell 4.3× as long as wide; M+CU1 largely sclerotized. Hind wing: M+CU:1-M:1r-m = 34:45:16; m-cu distinct, curved and unsclerotized.

***Legs***: Hind coxa rugose dorsally and remainder largely smooth; tarsal claws rather slender, evenly curved and longer than arolium (Fig. 86); length of femur, tibia and basitarsus of hind leg 5.7, 12.0, and 9.6× their width, respectively; hind leg densely setose; hind tarsus slender (Fig. 86) and slightly longer than tibia.

***Metasoma***: Length of first tergite 1.1× its apical width, its surface coarsely longitudinally costate-striate, its dorsal carinae converging and meeting submedially (Fig. 83); dorsope deep and medium-sized (Fig. 83); basal 0.7 of second tergite entirely coarsely longitudinally striate; remainder of metasoma smooth; third tergite in lateral view flat.

***Colour***: Black or blackish brown; mandible, palpi, clypeus and second tergite laterally pale yellowish; legs (but hind tibia and all tarsi infuscate or dark brown), tegulae and basal half of metasoma ventrally brownish yellow; apical half of metasoma dark brown ventrally; face yellowish brown; propleuron posteriorly, orbita and temple reddish brown; pterostigma, second and third tergites dark brown and most veins brown; wing membrane subhyaline.

### Phasmidiasta

Taxon classificationAnimaliaHymenopteraBraconidae

Wharton, 1980

59DE308D-5935-53A7-8361-754D84AC0A72

Figs 92–105

Phasmidiasta Wharton, 1980: 63; Belokobylskij 1998: 169, 294–296; Fischer 2006: 628. Type species (by original designation): Phasmidiasta lia Wharton, 1980 [holotype (CNC) examined].

#### Notes.

The biology of this Holarctic genus is uncertain. It is likely a parasitoid of xylophilous fly larvae (Wharton 1980). Only two species are known: *P. effecta* Belokobylskij, 1998, occurs in the Eastern Palaearctic region (reared from a cocoon in a bark beetle gallery (Belokobylskij 1998) and the type species in the Nearctic region. The Neotropical *P. ecuadorensis* Fischer, 2006, does not belong in *Phasmidiasta* and is transferred to *Neodiasta* van Achterberg, gen. nov. (see above) and *P. malaysiae* Fischer, 2006, was transferred to *Separatatus* by Yao et al. (2018a). The species remaining in *Phasmidiasta* are very similar and may be separated as follows:

**Table:** 

1	Scutellar sulcus laterally connected to posteriorly diverging and narrow oblique grooves (Fig. 95); ovipositor sheath 1.4–1.5? as long as metasoma and 0.6–0.7? as long as fore wing (Fig. 102); first metasomal tergite 1.2–1.4? as long as its apical width (Fig. 96); Nearctic (Canada)	***P. lia* Wharton, 1980**
–	Scutellar sulcus transverse, without narrow oblique grooves laterally; ovipositor sheath 1.1 ? as long as metasoma and 0.7 ? as long as fore wing; first tergite 1.5? as long as its apical width; Eastern Palaearctic (Far East Russia)	***P. effecta* Belokobylskij, 1998**

**Figures 92–105.**
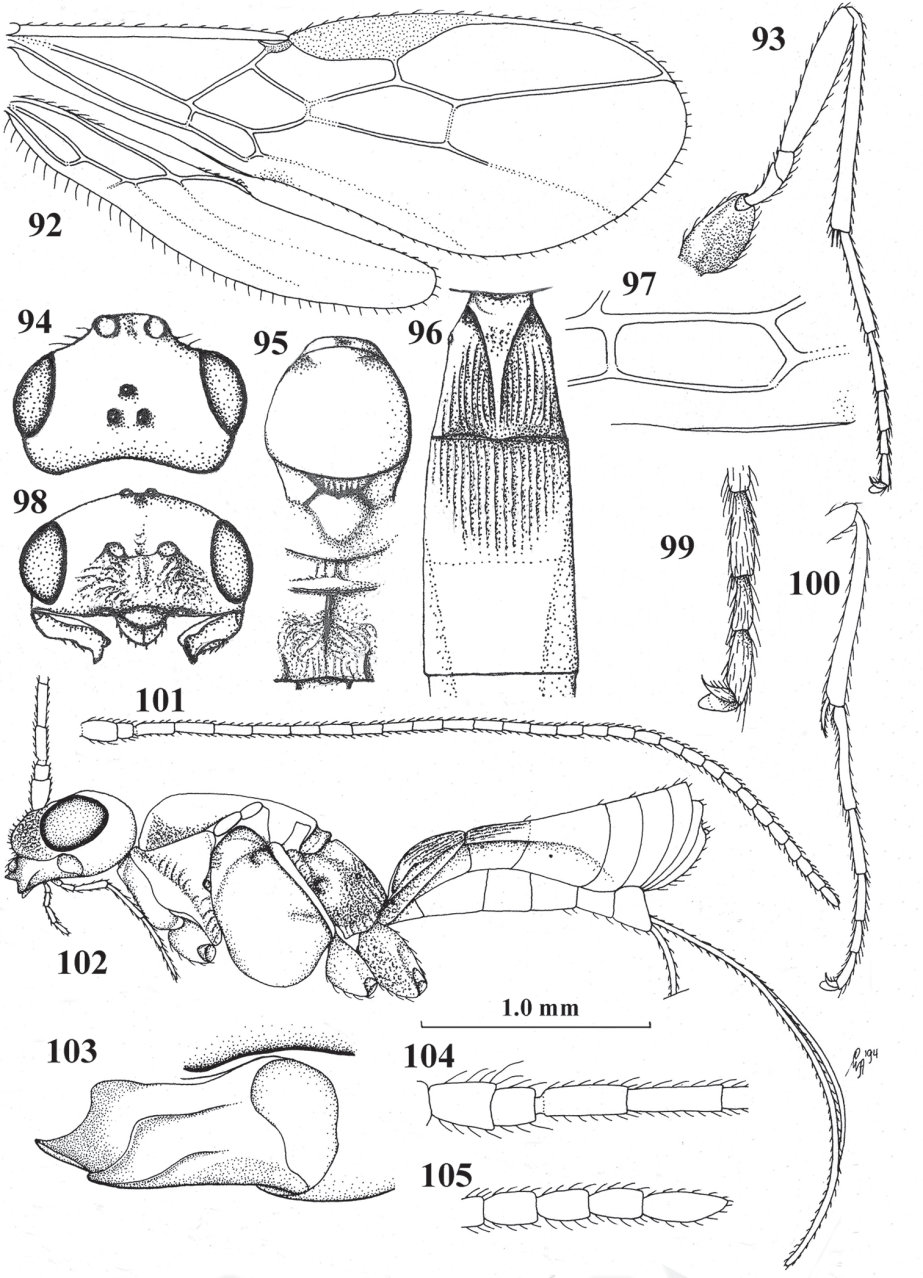
*Phasmalysia lia* Wharton, ♀, holotype **92** wings **93** hind leg **94** head, dorsal aspect **95** mesosoma, dorsal aspect **96** first–third metasomal tergites, dorsal aspect **97** first subdiscal cell of fore wing **98** head, anterior aspect **99** outer hind claw, lateral aspect **100** fore tibia and tarsus lateral aspect **101** antenna **102** habitus, lateral aspect **103** mandible, full view of first tooth **104** basal antennal segments **105** apical antennal segments.

### Senwot

Taxon classificationAnimaliaHymenopteraBraconidae

Wharton, 1983

C672011E-A0A0-57C9-B2AB-85CEE1DDBA81

Figs 106–114

Senwot Wharton, 1983: 277–279; Fischer 1991: 31 (redescription). Type species (by original designation): Senwot africanus Wharton, 1983 [holotype (AEI) was unavailable].

#### Notes.

A small genus of Afrotropical and Oriental species with unknown biology. The four species can be identified with the key by Yao et al. (2018b). Morphologically similar to *Bobekoides* and *Hylcalosia*, as shown by the shape of the mandible and clypeus, the genus differs mainly by the parallel-sided and more or less elongated pterostigma (in Asian spp. less than in African spp.).

**Figures 106–114.**
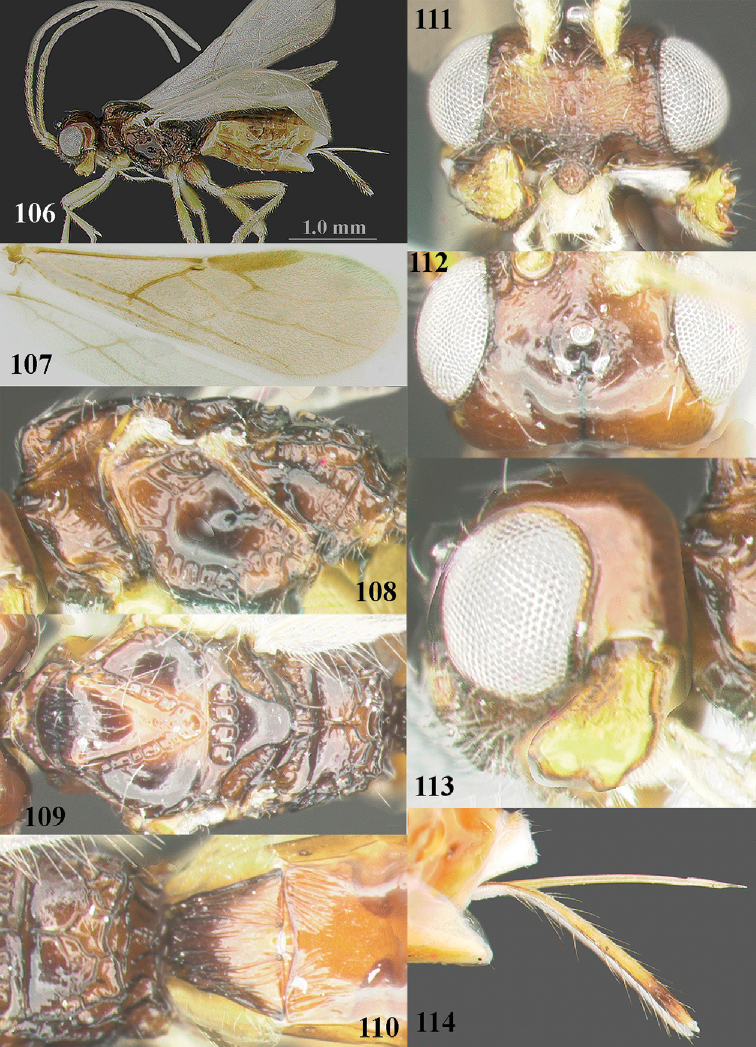
*Senwot yinxianggaoae* Yao, ♀, holotype **106** habitus, lateral aspect **107** wings **108** mesosoma, lateral aspect **109** mesosoma, dorsal aspect **110** propodeum, first–third metasomal tergites, dorsal aspect **111** head, anterior aspect **112** head, dorsal aspect **113** head, lateral aspect **114** ovipositor and its sheath, lateral aspect. Photos: J-L Yao.

**Figures 115–126.**
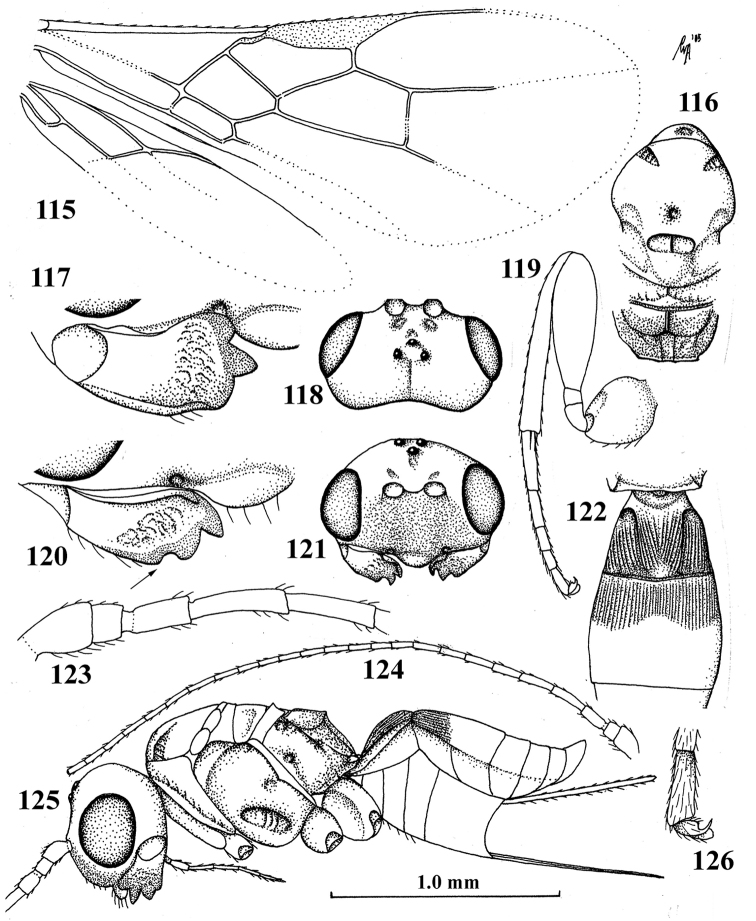
*Separatatus carinatus* Chen & Wu, ♀, holotype **115** wings **116** mesosoma, dorsal aspect **117** mandible, full view of first tooth **118** head, dorsal aspect **119** hind leg **120** mandible, full view of third tooth (fourth tooth arrowed) **121** head, anterior aspect **122** first–third metasomal tergites, dorsal aspect **123** basal antennal segments **124** antenna **125** habitus, lateral aspect **126** outer hind claw, lateral aspect.

## Supplementary Material

XML Treatment for Separatatus

XML Treatment for Parabobekoides

XML Treatment for Separatatus (Parabobekoides) yinshani

XML Treatment for Bobekia

XML Treatment for Bobekoides

XML Treatment for Hovalysia

XML Treatment for Hylcalosia

XML Treatment for Neodiasta

XML Treatment for Neodiasta ecuadorensis

XML Treatment for Phasmidiasta

XML Treatment for Senwot
